# Integrated CRISPR-Cas9 System-Mediated Knockout of IFN-γ and IFN-γ Receptor 1 in the Vero Cell Line Promotes Viral Susceptibility

**DOI:** 10.3390/ijms23158217

**Published:** 2022-07-26

**Authors:** Suyeon Kim, Aleksandra Nowakowska, Young Bong Kim, Ha Youn Shin

**Affiliations:** Department of Biomedical Science and Engineering, Konkuk University, Seoul 05029, Korea; 0924tndus123@naver.com (S.K.); szalonystudent@gmail.com (A.N.); kimera@konkuk.ac.kr (Y.B.K.)

**Keywords:** CRISPR-Cas9, interferon-gamma pathway, knockout, virus susceptibility

## Abstract

The current pandemic and the possible emergence of new viruses urgently require the rapid development of antiviral vaccines and therapeutics. However, some viruses or newly generated variants are difficult to culture in common cell types or exhibit low viral susceptibility in vivo, making it difficult to manufacture viral vector-based vaccines and understand host–virus interactions. To address these issues, we established new cell lines deficient in both type I and type II interferon responses, which are essential for host immunity and interference with virus replication. These cell lines were generated by developing an integrated CRISPR-Cas9 system that simultaneously expresses dual-guide RNA cassettes and Cas9 nuclease in a single plasmid. Using this highly efficient gene-editing system, we successfully established three cell lines starting from IFN-α/β-deficient Vero cells, deleting the single interferon-gamma (*IFNG*) gene, the IFNG receptor 1 (*IFNGR1*) gene, or both genes. All cell lines clearly showed a decrease in IFN-γ-responsive antiviral gene expression and cytokine production. Moreover, production of IFN-γ-induced cytokines remained low, even after HSV-1 or HCoV-OC43 infection, while expression of the receptor responsible for viral entry increased. Ultimately, knockout of IFN-signaling genes in these cell lines promoted cytopathic effects and increased apoptosis after viral infection up to three-fold. These results indicate that our integrated CRISPR-Cas9-mediated *IFNG*- and *IFNGR1*-knockout cell lines promote virus replication and will be useful in viral studies used to design novel vaccines and therapies.

## 1. Introduction

The coronavirus disease-19 (COVID-19) pandemic caused by severe respiratory syndrome coronavirus-2 (SARS-CoV2) infection continues to be a threat to global health. This unexpected pandemic has accelerated the development of antiviral vaccines and energized the therapeutics industry. Optimal manufacture of viral vector-based vaccines and in-depth viral studies require standardized in vitro and in vivo virus cultivation systems. One widely used mammalian cell line for mass production of viral vectors is the Vero cell line, derived from renal epithelial cells from the African green monkey [1]. This cell line is incapable of secreting the type I interferon family members, interferon-alpha (IFN-α) and IFN-beta (INF-β), which play important roles in antiviral activity [2]. The replication of a wide array of viruses, including influenza virus, Japanese encephalitis virus, and rabies virus, is facilitated in cells lacking genes encoding IFN-α and IFN-β [1,3]. However, not all viruses are easily cultured in the Vero cell line. In particular, the newly emergent SARS-CoV-2 and its variants, as well as human coronavirus OC43 (HCoV-OC43), do not replicate efficiently in vitro [4,5,6,7].

To further enhance the viral susceptibility of the Vero cell line, we sought to inhibit the IFN-gamma (IFN-γ) activation pathway, which is critical for both innate and adaptive immune responses of the host to bacterial and viral infections. One of the essential functions of IFN-γ, the only member of the type II interferon family, is to directly inhibit viral replication [8]. IFN-γ treatment directly inhibits in vivo replication of hepatitis B virus, measles virus, and murine cytomegalovirus (CMV), and in cooperation with IFN-β, inhibits SARS-CoV replication in vitro [9]. Cellular activation of the IFN-γ pathway is initiated by stimulation of the IFN-γ receptor (IFNGR) by IFN-γ [8]. IFNGR is a heterodimeric cell-surface receptor consisting of the ligand-binding IFN-γ receptor 1 (IFNGR1) and the accessory receptor IFNGR2. Binding of IFN-γ to IFNGR1 activates the associated Janus kinases, JAK1 and JAK2, which in turn phosphorylate a specific tyrosine residue in STAT1 (signal transducer and activator of transcription 1). Activated STAT1 homodimers translocate to the nucleus, where they induce a wide range of interferon-stimulated genes (ISGs), including interferon regulatory factor 1 (IRF-1), which mediate antiviral responses. IFN-γ and IFN-γ receptor proteins are encoded by *IFNG* and *IFNGR* genes, respectively. Mice lacking *IFNG* or *IFNGR* exhibit enhanced susceptibility to herpes simplex virus type 1 (HSV-1) as well as vaccinia virus and fail to exhibit IFN-γ-mediated antiviral responses to Sindbis virus [10]. Mice deficient in all three IFN genes—IFN-α, IFN-β, and IFN-γ—show increased susceptibility to lymphocytic choriomeningitis virus and vaccinia virus [11]. These observations suggest that suppressing IFN-γ expression in the IFN-α/β-deficient Vero cell line could enhance viral susceptibility in vitro.

To test this, we deleted *IFNG* and/or *IFNGR1* genes in the Vero cell line using CRISPR (clustered regularly interspaced short palindromic repeats)-Cas9 (CRISPR-associated protein 9) gene-editing technology, which was developed based on the CRISPR-Cas9 system originally identified as a microbial immune system that serves to eliminate invading viruses [12,13]. Further engineering of CRISPR-Cas9 into a simple two-component system consisting of guide RNA (gRNA) and Cas9 endonuclease enables gene editing in a wide variety of organisms [14]. Once gRNA recognizes its target DNA sequence, the associated Cas9 nuclease generates a double-stranded break with three base pairs (bp) upstream of a protospacer adjacent motif (PAM; NGG sequence), which results in degradation of the cleaved sites by exonucleases. Subsequently, the non-homologous end-joining DNA-repair system randomly repairs the damaged DNA strand, leaving nucleotide deletions in the target region. Intracellular expression of gRNA and Cas9 is typically achieved by the simultaneous introduction of each expression vector into the cells. To enhance the gene-editing efficiency, recent studies have often utilized a single plasmid that simultaneously expresses both gRNA and Cas9 nuclease [15,16,17]. Applying this strategy, we created an integrated CRISPR-Cas9 gene-editing platform consisting of dual gRNA cassettes and green fluorescent protein (GFP)-linked Cas9. We then used this integrated platform to successfully establish three different gene-edited IFN-α/β-deficient Vero cell lines: one lacking *IFNG*, one lacking *IFNGR1*, and one lacking both. Our results indicate that ablation of both IFN-γ and IFN-α/β pathways suppressed antiviral responses in vitro and further promoted viral replication. The *IFNG*/*IFNGR1*-knockout Vero cell line will facilitate studies designed to optimize antiviral vaccine production and virus analysis.

## 2. Results

### 2.1. Generation of IFNG- and IFNGR1-Knockout Cell Lines Using an Integrated CRISPR-Cas9 System

**Figure 1:**
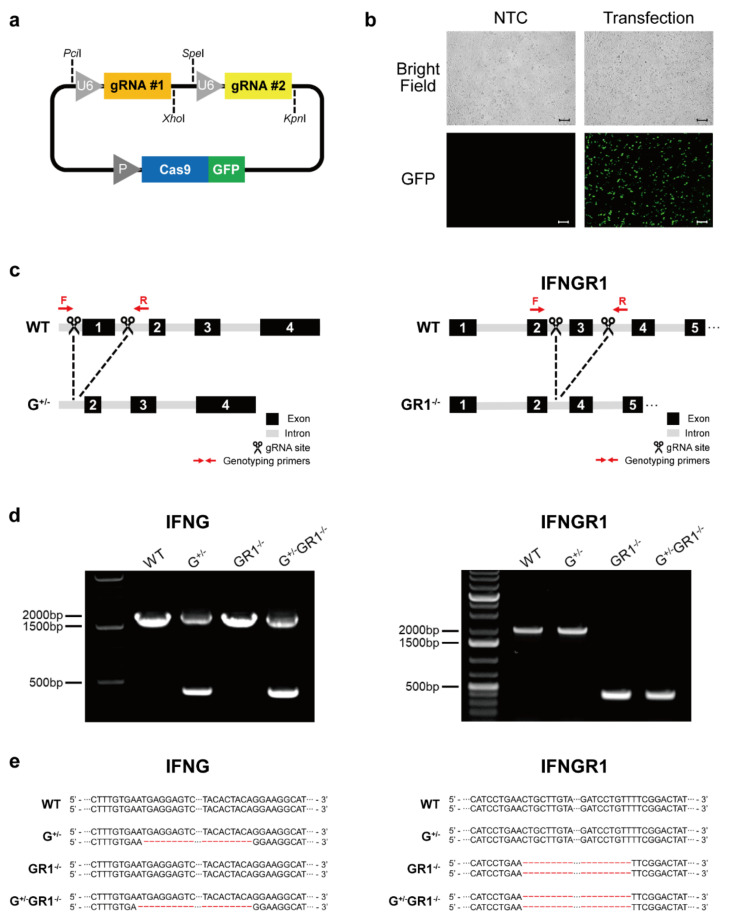
Generation of *IFNG*- and *IFNGR1*-knockout Vero cell lines using the integrated CRISPR-Cas9 system. (**a**) Schematic representation of an integrated CRISPR-Cas9 vector platform containing dual-guide RNA cassettes and GFP-linked Cas9 endonuclease. (**b**) Microscopic analysis of Vero cells transfected with integrated CRISPR-Cas9 expression plasmids. Fluorescent images were captured using a fluorescence microscope. Representative images are shown from independent triplicates. NTC, non-transfected control; GFP, green fluorescent protein. Scale bars: 10 μM. (**c**) Genomic loci of *IFNG* and *IFNGR1* targeted with CRISPR-Cas9. Exon 1 of *IFNG* and exon 3 of *IFNGR1*, respectively, were deleted in Vero cells. The annealing sites of genotyping primers are indicated by red arrows. (**d**) PCR analysis of targeted loci corresponding to deletion of exon 1 of the *IFNG* gene (left panel) and exon 3 of the *IFNGR1* gene (right panel). Double bands indicate the wild-type allele (upper band) and *IFNG*-knockout allele (lower band). (**e**) Deleted *IFNG* (left panel) and *IFNGR1* (right panel) sequences are shown for *IFNG*-heterozygous-knockout (G^+/−^), *IFNGR1*-homozygous-knockout (GR1^−/−^), and *IFNG/IFNGR1*-double-knockout (G^+/−^GR1^−/−^) Vero cell lines.

To ensure that both gRNA and Cas9 nucleases are delivered to the same cell, we generated a single plasmid containing both gRNA and Cas9 expression cassettes. We first constructed a single gRNA expression cassette by PCR amplification of the pUS-SH231 vector (Addgene) from the U6 promoter to the gRNA scaffold. For convenient cloning of any gRNA sequence into the CRISPR-Cas9 expression vector, we inserted *Pci*I- and *Xho*I-restriction-enzyme sites at each end of the gRNA cassette. The Cas9 expression vector backbone was prepared by removing original gRNA sequences in pUS-SH231, followed by insertion of this single gRNA cassette into the *Pci*I- and *Xho*I-digested Cas9 expression vector backbone. For simultaneous deletion of multiple sites, we further inserted tandem repeats of the gRNA scaffold containing *Spe*I and *Kpn*I sites into the CRISPR-Cas9 expression vector, which we termed the integrated CRISPR-Cas9 vector platform (Figure 1a). This vector contains a GFP reporter adjacent to the Cas9 protein, enabling us to easily estimate the transfection efficiency of this plasmid in cells. After confirming that this newly established, integrated CRISPR-Cas9 system was successfully expressed in the Vero cell line (Figure 1b), we inserted gRNA targeting exon 1 of the *IFNG* or exon 3 of the *IFNGR1* gene into this platform (Figure 1c). We ultimately obtained three distinct cell lines: one with heterozygous deletion of *IFNG* (G^+/−^), one with homozygous deletion of *IFNGR1* (GR^−/−^), and one with combined deletion of *IFNG* and *IFNGR1* (G^+/−^GR^−/−^) (Figure 1d). Unfortunately, we were unable to obtain a homozygous *IFNG*-knockout Vero cell line, possibly because triple knockout of *IFNA*, *IFNB*, and *IFNG* genes is detrimental to cells. The specific sequences deleted in each cell line were examined by PCR-based genotyping, followed by Sanger sequencing (Figure 1e, Supplementary Figure S1, and Supplementary Table S1). Taken together, these results confirmed that our integrated CRISPR-Cas9 system successfully functioned in generating specific gene deletions and was efficient for establishing gene-editing cell lines.

### 2.2. Downregulation of Immune-Related Genes in IFNG- and IFNGR1-Deficient Cells

**Figure 2:**
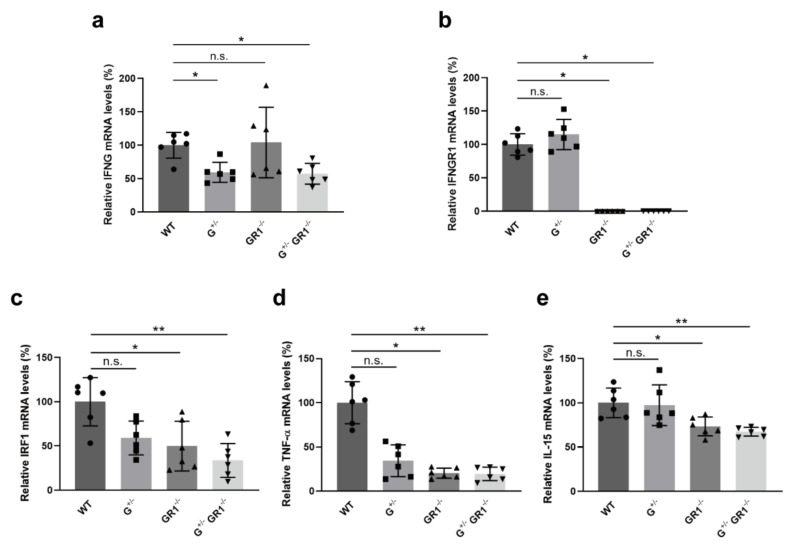
Downregulation of IFN-γ-responsive genes in *IFNG*- and *IFNGR1*-knockout Vero cell lines. (**a**) Relative *IFNG* expression levels in G^+/−^, GR1^−/−^, and G^+/−^GR1^−/−^ cell lines. IFNG mRNA levels in knockout cell lines were normalized to those of wild-type cells. Heterozygous knockout of *IFNG* in Vero cells reduced *IFNG* expression level by ~50% in G^+/−^ and G^+/−^GR1^−/−^ cell lines. Results are shown as means ± SD of six independent replicates (applies to all gene-expression data, * *p* < 0.05; ** *p* < 0.01; n.s., not significant). (**b**) Relative *IFNGR1* expression levels in G^+/−^, GR1^−/−^, and G^+/−^GR1^−/−^ cell lines. The *IFNGR1* gene was completely silenced in GR1^−/−^ and G^+/−^GR1^−/−^ cell lines. (**c**–**e**) Relative expression levels of *IRF-1* (**c**), *TNF-α* (**d**), and *IL-15* (**e**) in G^+/−^, GR1^−/−^, and G^+/−^GR1^−/−^ cell lines. Ablation of *IFNG* and *IFNGR1* genes downregulated the transcription of genes related to the IFN-γ pathway.

Next, we assessed the expression levels of *IFNG* and *IFNGR1* genes in knockout cell lines. As expected, heterozygous knockout of the *IFNG* gene resulted in approximately a 50% decrease in IFNG mRNA expression levels in G^+/−^ and G^+/−^GR1^−/−^ cell lines compared with wild-type cells (Figure 2a). Homozygous knockout of *IFNGR1* completely silenced the *IFNGR1* gene in GR1^−/−^ and G^+/−^GR1^−/−^ cell lines (Figure 2b). We further examined the expression levels of several IFN-γ-responsive genes. IRF-1, a transcription factor directly induced by IFN-γ stimulation [8] that regulates genes involved in innate and adaptive immunity [18,19], can also be induced by different stimuli in various cell types. Once IFN-γ activates the JAK/STAT pathway, STAT1 homodimers bind to the *IRF1* promoter and induce expression of the corresponding gene [8]. We found that deletion of *IFNG* or *IFNGR1* in the Vero cell line clearly downregulated *IRF-1* expression levels by ~50% compared to that in wild-type cells and by 30% in the *IFNG*/*IFNGR1*-double-knockout cell line (Figure 2c). IRF-1 can be recruited to the promoter of various genes to regulate immune responses, apoptosis, and tumor suppression. For example, IRF-1 regulates the transcription of tumor necrosis factor alpha (TNF-α), which plays a pivotal role in antiviral responses [20]. *TNF-α* expression levels in our GR1^−/−^ and G^+/−^GR1^−/−^ cell lines were also significantly reduced by ~20% compared to that in wild-type cells (Figure 2d). Interleukin-15 (IL-15), another cytokine induced by IFN-γ receptor signaling [21], primarily functions in host defense against viral infection [22]. Like the case for TNF-α, IFN-γ-induced IRF-1 binds to the promoter of the *IL-15* gene to promote transcription of the corresponding gene and production of IL-15 protein. Our results revealed that IL-15 mRNA levels were reduced by ~30% in GR1^−/−^ and G^+/−^GR1^−/−^ cell lines (Figure 2e). However, the *IL-15* expression level in the G^+/−^ cell line was comparable to that of wild-type cells, indicating that the production of IL-15 is more dependent on the IFN-γ receptor. Taken together, our results indicate that *IFNG* and/or *IFNGR* expression levels were reduced as expected in G^+/−^, GR1^−/−^, and G^+/−^GR1^−/−^ cell lines. The absence of *IFNG* and *IFNGR1* further downregulated IFN-γ-responsive transcription factors and cytokines that play a critical role in host immunity against viral infection, events that are independent of the IFN-α/β pathway.

### 2.3. Enhanced Cytopathic Effect of HSV-1 Infection in IFNG- and IFNGR1-Knockout Vero Cells

We further examined whether an *IFNG* or *IFNGR1* deficiency enhances the viral susceptibility of Vero cells. We first infected *IFNG*- and *IFNGR1*-knockout cell lines with HSV-1, a double-stranded DNA virus that can be propagated in the Vero cell line [23], at a multiplicity of infection (MOI) of 0.1 for 72 h and then observed the cytopathic effects daily under a light microscope. A previous study showed that IFN-γ and IFN-β treatment synergized to inhibit the replication of HSV-1 in vitro and in vivo, demonstrating that treatment of Vero cells with either IFN-γ or IFN-β decreased virus replication by less than ~20-fold, whereas treatment with both IFN-γ and IFN-β reduced HSV-1 replication by ~1000-fold [9]. Moreover, pretreatment of mouse eyes with both IFN-γ and IFN-β significantly prevented HSV-1 replication and viral pathogenesis. Accordingly, interfering with both IFN-γ and IFN-α/β pathways may promote HSV-1 replication.

**Figure 3:**
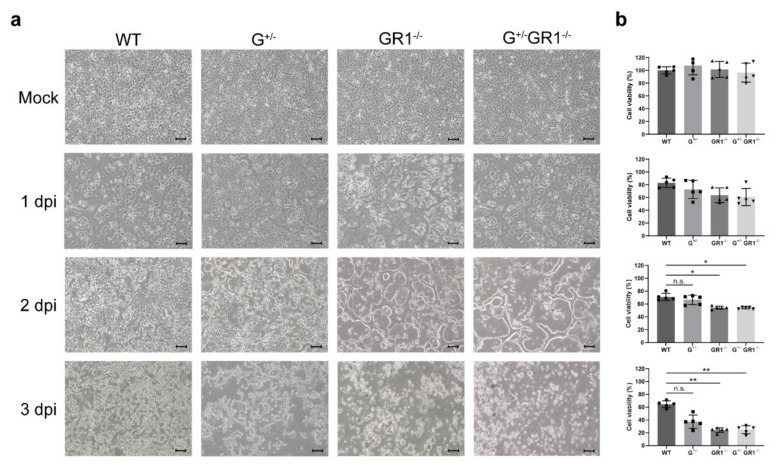
Susceptibility of *IFNG*- and *IFNGR1*-knockout Vero cell lines to HSV-1 infection. (**a**) The cytopathic effects of HSV-1 infection in G^+/−^, GR1^−/−^, and G^+/−^GR1^−/−^ cell lines in comparison to wild-type cells. Cytopathic effects were monitored daily with a light microscope, and photographs of cell monolayers were acquired every 24 h after infection. Representative images are shown from independent triplicates. Scale bars: 10 μM (applies to all microscopic images). (**b**) Relative viability of G^+/−^, GR1^−/−^, and G^+/−^GR1^−/−^ cell lines after HSV-1 infection compared with that in wild-type cells. Cell viability was determined by MTT assay. Cell viability in each cell line was normalized to that of uninfected wild-type controls. Results are shown as means ± SD of five independent replicates (applies to all MTT assays, * *p* < 0.05; ** *p* < 0.01; n.s., not significant).

HSV-1 typically induces cytopathic effects at least 1 to 3 days after infection [23]. In our study, initial signs of cytopathic effects, such as ballooning of infected cells, were evident in GR1^−/−^ and G^+/−^GR1^−/−^ cell lines 1 day after HSV-1 infection, a time when uninfected control cells exhibited a normal morphology (Figure 3a). Two days after infection, all three cell lines (G^+/−^, GR1^−/−^, and G^+/−^/GR1^−/−^) started to show morphological changes, including rounding of infected cells and fusion with adjacent cells. We also performed MTT (3-[4,5-dimethylthiazol-2-yl]-2,5 diphenyl tetrazolium bromide) assays to measure cell viability and assess its correlation with cytopathic effects (Figure 3b). Cell viability, measured daily until 3 days after HSV-1 infection, was found to be proportional to the observed cytopathic effects. On day 3 after viral infection, all three cell lines—G^+/−^, GR1^−/−^, and G^+/−^/GR1^−/−^—exhibited severe cytopathic effects, including cell swelling, formation of cell clumps, and extensive cell detachment (Figure 3a). Consistent with this, more than 80% of cells in all three knockout cell lines died (Figure 3b). The viability of infected GR1^−/−^ and G^+/−^GR1^−/−^ cell lines was almost three-fold less than that of the infected wild-type Vero cell line. We also carried out endpoint dilution assays to measure virus titer in wild-type and G^+/−^GR1^−/−^ cell lines (Supplementary Figure S2). Quantification of HSV-1 collected 3 days post-infection from supernatants of the G^+/−^GR1^−/−^ cell line showed a TCID_50_ (50% tissue culture infective dose) value 10-fold higher than that of wild-type cells. Taken together, our data indicate that viral replication is much more active in the absence of all three IFNs (IFN-α, -β, and -γ)

### 2.4. Downregulation of the Immune Response to HSV-1 Infection in IFNG- and IFNGR1-Knockout Vero Cell Lines

**Figure 4:**
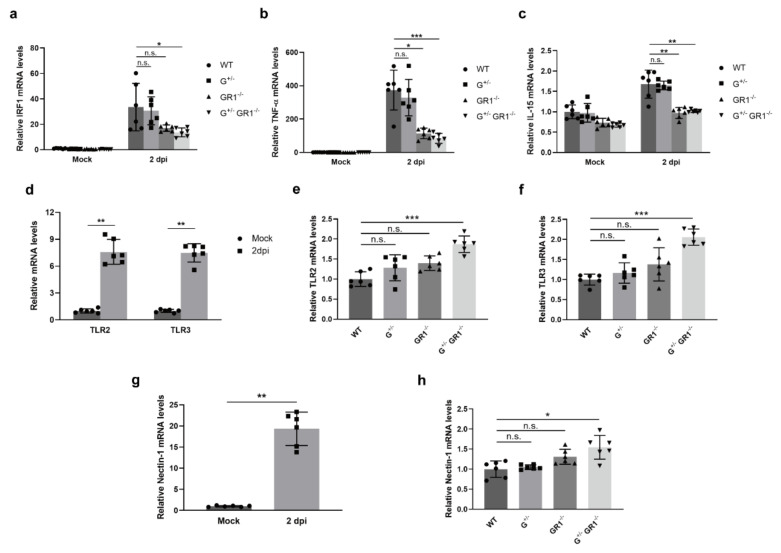
HSV-1 infection causes downregulation of IFN-γ-induced cytokines coupled with an increase in the receptor responsible for HSV-1 entry in *IFNG*- and *IFNGR1*-knockout Vero cell lines. (**a**–**c**) Relative *IRF-1* (**a**), *TNF-α* (**b**), and *IL-15* (**c**) expression levels in G^+/−^, GR1^−/−^, and G^+/−^GR1^−/−^ cell lines after HSV-1 infection. IRF-1, TNF-α, and IL-15 mRNA levels in knockout cell lines were normalized to those of uninfected wild-type controls. Results are shown as means ± SD of six independent replicates (applies to all gene-expression data, * *p* < 0.05; ** *p* < 0.01; *** *p* < 0.001; n.s., not significant). (**d**) Relative expression levels of *TLR2* and *TLR3* in wild-type Vero cells 2 days after HSV-1 infection. TLR2 and TLR3 mRNA levels were normalized to those of uninfected controls. (**e**,**f**) Relative expression levels of *TLR2* (**e**) and *TLR3* (**f**) in G^+/−^, GR1^−/−^, and G^+/−^GR1^−/−^ cell lines after HSV-1 infection. TLR2 and TLR3 mRNA levels in HSV-1 infected G^+/−^, GR1^−/−^, and G^+/−^GR1^−/−^ cell lines were normalized to those of infected wild-type cells. (**g**) Relative expression level of the viral-entry-associated receptor, nectin-1, in wild-type Vero cells 2 days after HSV-1 infection. Nectin-1 mRNA level was normalized to that of uninfected controls. (**h**) Relative expression levels of *nectin-1* in G^+/−^, GR1^−/−^, and G^+/−^GR1^−/−^ cell lines after HSV-1 infection. Nectin-1 mRNA levels in HSV-1-infected G^+/−^, GR1^−/−^, and G^+/−^GR1^−/−^ cell lines were normalized to those of infected wild-type cells.

Next, we investigated the molecular mechanisms underlying the enhanced viral susceptibility of Vero cell lines lacking *IFNG* or *IFNGR1* genes. We first examined expression levels of HSV-1-infection-induced, IFN-γ-responsive genes in wild-type and knockout cell lines. As expected, HSV-1 infection induced a substantial increase in IRF-1, TNF-α, and IL-15 mRNA levels in the wild-type Vero cell line, increasing *IRF-1* up to 30-fold 2 days after viral infection (Figure 4a–c). However, in GR1^−/−^ and G^+/−^GR1^−/−^ cell lines, HSV-1-infection-induced *IRF-1* and *IL-15* expression levels were ~two-fold less and *TNF-α* levels were almost four-fold reduced compared with wild-type and G^+/−^ cell lines (Figure 4a–c). Expression levels of *IRF-1*, *TNF-α*, and *IL-15* in the infected G^+/−^ cell line were not significantly different from that in infected wild-type cells, possibly reflecting compensation of IFN-γ production by the remaining *IFNG* allele. These results indicate that the absence of either *IFNGR1* or *IFNG*/*IFNGR1* suppresses the expression of IFN-γ-induced transcription factors and cytokines even after HSV-1 infection, which may be beneficial for viral replication.

Toll-like receptors (TLRs) are pattern recognition receptors that recognize specific patterns of ‘non-self’ molecules, such as invading pathogens, and trigger host innate immunity [24]. HSV-1 is primarily recognized by TLR2 and TLR3, which subsequently recruit adaptor proteins to activate signaling pathways that produce inflammatory cytokines and interferons [25,26]. A previous study showed that TLR2 and TLR3 mRNA levels are increased by HSV-1 infection in Vero cells [27]. Indeed, we also found that *TLR2* and *TLR3* expression levels were induced up to eight-fold in wild-type Vero cells after HSV-1 infection (Figure 4d). Interestingly, expression levels of *TLR2* and *TLR3* were increased up to two-fold in the G^+/−^GR1^−/−^ cell line compared with infected wild-type cells, indicating greater production of virus in *IFNG*- and *IFNGR1*-knockout cell lines (Figure 4e,f). However, owing to the inability to activate the IFN-α/β and IFN-γ pathways in *IFNG*/*IFNGR*-knockout Vero cell lines, levels of the cytokines, TNF-α, and IL-15 remained low (Figure 4b,c), which may aid in virus spread. We further explored whether the enhanced susceptibility of *IFNG*- and *IFNGR1*-knockout cell lines to HSV-1 infection was associated with increased viral entry into cells. Nectin-1 is a cellular receptor that plays a key role in mediating HSV-1 entry into cells [28]. Attachment of viral glycoprotein D to nectin-1 on the cell surface leads to the formation of glycoprotein complexes and induces fusion of the viral envelope with the plasma membrane, thereby allowing penetration of the viral capsid into host cell. Several studies have shown that nectin-1 can also help spread the virus by virtue of its increased expression levels and accumulation at cell junctions in HSV-infected cells [29,30]. Indeed, *nectin-1* expression level increased ~20-fold after HSV-1 infection in wild-type Vero cells (Figure 4g). Intriguingly, *nectin-1* expression levels were increased up to 1.5-fold in infected *IFNG*- and *IFNGR1*-knockout Vero cell lines compared with infected wild-type Vero cells (Figure 4h), indicating that viral entry through nectin-1 was potentiated in *IFNG*- and *IFNGR1*-knockout Vero cell lines. Taken together, our data suggest that abrogation of the IFN-γ pathway in the Vero cell line may aid HSV-1 replication by inhibiting IFN-γ-induced cytokines and upregulating the receptor responsible for cellular entry of HSV-1.

### 2.5. Enhanced Cytopathic Effects in IFNG- and IFNGR1-Knockout Vero Cells after HCoV-OC43 Infection

We further examined whether *IFNG*- and *IFNGR1*-knockout cell lines show enhanced susceptibility to infection by different types of viruses besides HSV-1. To this end, we infected each cell line with human coronavirus OC43 (HCoV-OC3; MOI = 0.1), an enveloped, positive-sense, single-stranded RNA member of the *Betacoronavirus* genus responsible for causing the common cold [31]. Previous studies have shown that HCoV-OC43 rarely causes clear cytopathic effects in infected cell lines; thus, typical plaque assays and TCID_50_ assays that monitor cytopathic effects are not suitable for virus titration [6,32]. Thus, we tested whether HCoV-OC43 infection induces significantly greater cytopathic effects in *IFNG*- or *IFNGR1*-knockout Vero cell lines by comparing cell morphology.

**Figure 5:**
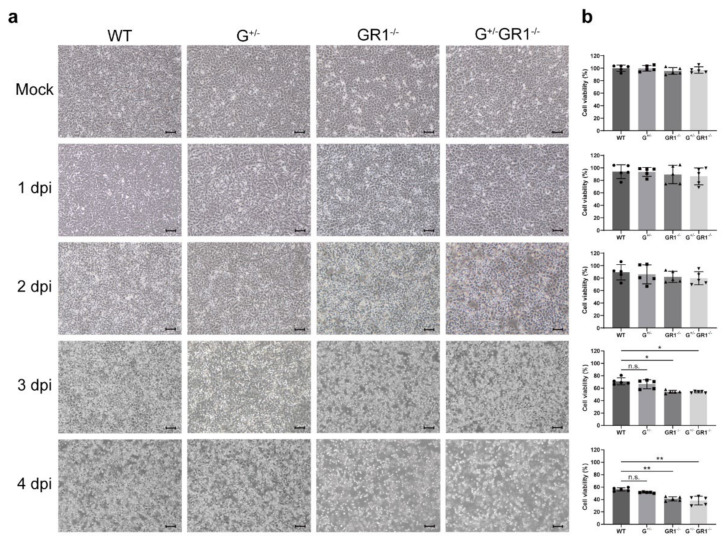
Susceptibility of *IFNG*- and *IFNGR1*-knockout Vero cell lines to infection by HcoV-OC43 virus. (**a**) Microscopic analysis of cytopathic effects in G^+/−^, GR1^−/−^, and G^+/−^GR1^−/−^ cell lines infected with HCoV-OC43 compared with infected wild-type cells. Cytopathic effects were monitored daily using a light microscope, and photographs of cell monolayers were captured every 24 h after infection. Representative images are shown from independent triplicates. Scale bars: 10 μM (applies to all images). (**b**) Viability of G^+/−^, GR1^−/−^, and G^+/−^GR1^−/−^ cell lines after HCoV-OC43 infection compared with that of wild-type cells. Cell viability, assessed by MTT assay, was normalized to that of uninfected wild-type controls. Results are shown as means ± SD of five independent replicates (* *p* < 0.05; ** *p* < 0.01; n.s., not significant).

In the wild-type Vero cell line, cell morphology was comparable to that of uninfected cells until 3 days post-infection, and only partial cell detachment occurred 4 days after HCoV-OC43 infection (Figure 5a). Although G^+/−^, GR1^−/−^, and G^+/−^GR1^−/−^ cell lines did not show clear cytopathic effects, such as cell morphological changes, 1 day after infection, cells exhibiting partial detachment began to appear in GR1^−/−^ and G^+/−^GR1^−/−^ cell lines 2 days after HCoV-OC43 infection. Then, 3 days after infection, more profound cytopathic effects, such as cell rounding and detachment, were observed in GR1^−/−^ and G^+/−^GR1^−/−^ cell lines, and extensive cell detachment occurred at 4 days post-infection. We also assessed the correlation between cytopathic effects observed microscopically and cell viability, determined by MTT assay (Figure 5b). Consistent with visual readouts, the number of viable wild-type Vero cells decreased by only ~40% after 4 days of HCoV-OC43 infection, whereas the number of GR1^−/−^ and G^+/−^GR1^−/−^ cells had already decreased by 50% after 3 days of viral infection. At 4 days post-infection, more than 60% of cells of GR1^−/−^ and G^+/−^GR1^−/−^ cell lines had died, and their viability was significantly lower than that of infected wild-type Vero cells. Virus titers obtained in the G^+/−^GR1^−/−^ cell line consistently trended higher than that in the infected wild-type Vero cell line during the first 3 days of viral infection (Supplementary Figure S3). These data indicate that the absence of *IFNG* and *IFNGR* in the newly generated *IFNG/IFNG*-knockout cell line clearly promoted HcoV-OC43 replication, which resulted in rapid cytopathic effects and increased apoptosis.

### 2.6. Downregulation of the Immune Response to Coronavirus Infection in IFNG- and IFNGR1-Knockout Vero Cell Lines

**Figure 6:**
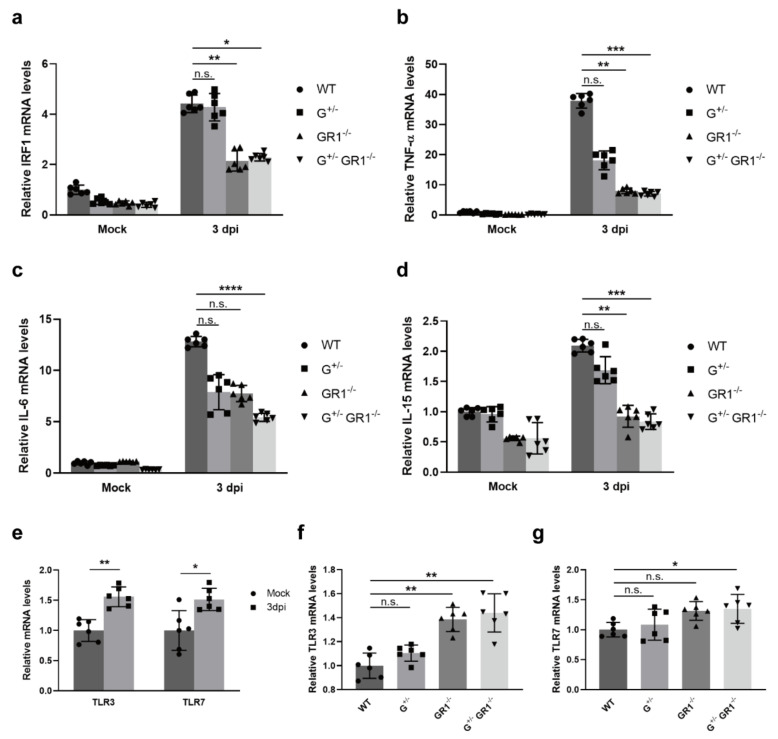
Downregulation of IFN-γ-induced cytokines in *IFNG*- and *IFNGR1*-knockout Vero cell lines upon HCoV-OC43 infection. (**a**–**d**) Relative *IRF-1* (**a**), *TNF-α* (**b**), *IL-6* (**c**), and *IL-15* (**d**) expression levels in G^+/−^, GR1^−/−^, and G^+/−^GR1^−/−^ cell lines after HCoV-OC43 infection. IRF-1, TNF-α, IL-6, and IL-15 mRNA levels in knockout cell lines were normalized to those of uninfected wild-type controls. Results are shown as means ± SD of six independent replicates (applies to all gene-expression data, * *p* < 0.05; ** *p* < 0.01; *** *p* < 0.001; **** *p* < 0.0001; n.s., not significant). (**e**) Relative expression levels of virus recognition receptors in the Vero cell line after HCoV-OC43 infection. TLR3 and TLR7 mRNA levels in HCoV-OC43-infected Vero cells were normalized to those in uninfected controls. (**f**,**g**) Relative expression levels of *TLR3* (**f**) and *TLR7* (**g**) in G^+/−^, GR1^−/−^, and G^+/−^GR1^−/−^ cell lines after HCoV-OC43 infection. TLR3 and TLR7 mRNA levels in HCoV-OC43-infected G^+/−^, GR1^−/−^, and G^+/−^GR1^−/−^ cell lines were normalized to those of infected wild-type cells.

Lastly, we investigated the molecular mechanism underlying the increased susceptibility to infection by HCoV-OC43 in *IFNG*- and *IFNGR1*-knockout cell lines. Several studies have shown that IFN-γ is upregulated upon HCoV-OC43 infection [33,34]. In our study, we also observed an increase in *IFNG* (up to six-fold) and *IFNGR1* (up to three-fold) expression levels in HCoV-OC43-infected wild-type Vero cells (Supplementary Figure S4a,b). However, *IFNG* levels were increased only ~three-fold in G^+/−^ and G^+/−^GR1^−/−^ cell lines upon viral infection, and *IFNGR1* levels remained completely unchanged in infected GR1^−/−^ and G^+/−^GR1^−/−^ cell lines. Next, we examined the expression levels of the IFN-γ-responsive antiviral genes, *IRF-1* and *TNF-α*. A previous study showed that *TNF-α* expression levels in vitro are upregulated by HCoV-OC43 infection or IFN-γ treatment [35]. Consistent with this, we found that mRNA levels of IRF-1, which mediates the transcription of TNF-α, increased more than four-fold after HCoV-OC43 infection in the wild-type Vero cell line (Figure 6a). TNF-α mRNA levels subsequently increased ~40-fold in these virus-infected wild-type cells (Figure 6b). In the G^+/−^ cell line, *IRF-1* expression levels were comparable to those in wild-type cells upon HCoV-OC43 infection. However, *IRF-1* expression levels were ~two-fold lower in GR1^−/−^ and G^+/−^GR1^−/−^ cell lines after viral infection. Subsequent *TNF-α* expression levels were two-fold lower in the G^+/−^ cell line and five-fold lower in GR1^−/−^ and G^+/−^GR1^−/−^ cell lines than in infected wild-type cells, indicating that IRF-1-mediated TNF-α production remained low in Vero cells lacking *IFNG* and *IFNGR1*, even after HCoV-OC43 infection. We further investigated expression levels of the IFN-γ-associated inflammatory cytokines, IL-6 and IL-15, following HCoV-OC43 infection in each cell line. A previous study showed that IL-6 expression in astrocytes is induced up to five-fold by IFN-γ treatment or HCoV-OC43 infection [34]. Our data also showed that *IL-6* expression levels were upregulated by up to 13-fold in wild-type Vero cells after HCoV-OC43 infection compared with uninfected controls (Figure 6c). However, IL-6 mRNA levels decreased by ~two-fold in G^+/−^, GR1^−/−^, and G^+/−^GR1^−/−^ cell lines compared with infected wild-type cells. Similarly, IL-15 levels were increased ~two-fold upon HCoV-OC43 infection in wild-type cells, while remaining unchanged in GR1^−/−^ and G^+/−^GR1^−/−^ cell lines (Figure 6d). These data indicate that the expression of IFN-γ-responsive transcription factors and cytokines remained low even after HCoV-OC43 infection in *IFNG*- and *IFNGR1*-knockout cell lines, potentially facilitating viral replication.

We also examined whether increased virus production in *IFNG*- and *IFNGR1*-knockout Vero cell lines caused the induction of TLR family members. Our data showed that both TLR3 and TLR7, which are known to recognize HCoV-OC43 invasion [35], were induced by more than 1.5-fold upon HCoV-OC43 infection in wild-type Vero cells (Figure 6e). As expected, TLR3 and TLR7 mRNA levels were increased up to 1.4-fold in GR1^−/−^ and G^+/−^GR1^−/−^ cell lines after HCoV-OC43 infection compared with infected wild-type cells (Figure 6f,g). However, levels of the cytokines TNF-α, IL-6, and IL-15 remained low owing to the inhibition of IFN-α/β and IFN-γ pathways. Taken together, our data suggest that the downregulation of IFN-γ-responsive antiviral genes promotes HCoV-OC43 replication in *IFNG*- and *IFNGR1*-knockout cell lines.

## 3. Discussion

The recent global pandemic has highlighted the need for rapid development of vaccines and antiviral treatments. One of the essential factors necessary for facilitating the manufacturing of viral vector-based vaccines or for research on unknown viral strains is a well-established in vitro or in vivo virus-culture system. Vero is among FDA-approved cell lines for manufacturing viral vector-based vaccines [1], but not all viruses replicate efficiently in this cell line. To address this issue, we modified the Vero cell line by deleting *IFNG* and *IFNGR1* genes to disrupt the IFN-γ activation pathway, which plays an important role in the antiviral response. We hypothesized that ablation of *IFNG* and *IFNGR1* in the Vero cell line would block IFN-α/β and IFN-γ activation pathways, thereby suppressing the antiviral response and promoting viral replication. To establish gene-edited cell lines, we created an integrated CRISPR-Cas9 vector platform that simultaneously expresses a dual gRNA cassette and a GFP-linked Cas9 nuclease. Separate expression of gRNA and Cas9 nuclease in cells often results in low gene-editing efficiency, leading to the failure to obtain gene-edited cell lines. Using the integrated CRISPR-Cas9 system, we successfully established three cell lines lacking *IFNG*, *IFNGR1*, or both genes.

Removal of *IFNG* and *IFNGR1* genes clearly inhibited the IFN-γ activation pathway and suppressed the transcription of the IFN-γ-inducible antiviral cytokines, TNF-α, and IL-15. This inhibitory response was more evident in the GR1^−/−^ and G^+/−^GR1^−/−^ cell lines, as the G^+/−^ cell line still carries a wild-type *IFNG* allele capable of compensating for IFN-γ secretion. Once the wild-type Vero cell line is infected with a virus such as HSV-1 or HCoV-OC43, the expression of *IFNG* and *IFNGR1* increases, followed by upregulation of IFN-γ-responsive genes. However, *IFNGR1*- and *IFNG/IFNGR1*-deficient cell lines maintained relatively low levels of IFN-γ-responsive cytokines upon HSV-1 or HCoV-OC43 infection. The cell line carrying a heterozygous deletion of *IFNG* expressed similar levels of cytokines as wild-type controls, possibly due to the remaining *IFNG* allele capable of secreting IFN-γ. Consequently, inhibition of antiviral responses further increased viral replication and promoted cytopathic effects and apoptosis. Increased viral replication was directly demonstrated by the higher virus titers obtained from *IFNG*- and *IFNGR1*-knockout cell lines and indirectly supported by the increase in TLR levels essential for the recognition of HSV-1 or HCoV-OC43. We have also noted that HSV-1 is more sensitive to the IFN-γ signaling pathway than HCoV-OC43. This may be due to the differences in the degree of contributions of IFN-γ to the innate immune response to these viruses. Previous studies have shown that IFN-γ plays a critical role in viral clearance by synergizing with IFN-α/β after HSV infection in vitro and in vivo [9,36,37], whereas IFN-γ and IFN-β were rarely induced after HCoV-OC43 infection in vitro [4]. Consistently, our data revealed that *IFN-γ* expression levels were induced over 110-fold upon HSV-1 infection in Vero cells, but only increased 6-fold upon HCoV-OC43 infection (Supplementary Figure S5). Cytopathic effects were also delayed after HCoV-OC43 infection compared to the HSV-1 infection (Figure 3 and Figure 5), possibly due to the mild innate response for an extended period. These results suggest that since HCoV-OC43 is a weaker inducer of IFN-γ than HSV-1, inactivation of the IFN-γ pathway may have a relatively less effect on HCoV-OC43 infection.

Our results clearly showed that IFN-γ-responsive genes, including *IRF-1*, *TNF-α*, and *IL-15*, were downregulated in the absence of *IFNG* and *IFNGR1*. These downregulated genes remained silent after infection with HSV-1 or HCoV-OC43, in association with an increase in the expression of receptor genes involved in viral entry. As a consequence, cytopathic effects were accelerated, and apoptosis was increased. Here, we used HSV-1 and HCoV-OC43 as representative DNA and RNA viruses to evaluate the viral susceptibility of *IFNG*- and *IFNGR1*-knockout Vero cell lines. In future studies, it will be interesting to characterize *IFNG*/*IFNGR1*-knockout Vero cells by testing other types of viruses, including non-enveloped and enveloped viruses, to gain insight into the optimal use of these cell lines. In addition, a comparison of global gene-expression profiles of infected and uninfected cell lines through RNA-sequencing analyses would provide further detail into the underlying mechanisms of enhanced viral replication in *IFNG*/*IFNGR1*-knockout Vero cells. Collectively, the data presented in the current study clearly demonstrate that our integrated CRISPR-Cas9 system is useful for generating gene-edited cell lines and, further, that *IFNG*/*IFNGR1*-knockout Vero cell lines are suitable for virus propagation and viral analyses.

## 4. Materials and Methods

### 4.1. Integrated CRISPR-Cas9 Vector System

The plasmid pUS-SH231 (Addgene, Cambridge, MA, USA, #115150) was used to generate a single plasmid vector that simultaneously expresses dual gRNAs and the GFP-tagged *Streptococcus pyogenes* Cas9 (SpCas9) endonuclease. pUS-SH231 was used as a backbone plasmid after removing the original sequence between the human U6 promoter and gRNA scaffold. The gRNA scaffold of the modified pUS-SH231 vector was obtained by PCR amplifying from the U6 promoter to the first gRNA cassette using targeted primers containing *Pci*I and *Xho*I site overhangs at each end and inserting this amplified segment into the *PciI*-and *XhoI*-digested pUS-SH231 vector. The second gRNA cassette was PCR amplified using primers containing *Spe*I and *Kpn*I site overhangs at each end and introduced into the *Spe*I- and *Kpn*I-digested pUS-SH231 vector containing a single gRNA cassette. Introduction of restriction-enzyme sites at each end of gRNA cassettes enables the easy insertion of any given gRNA into the vector platform.

### 4.2. Design of gRNAs and the Construction of IFNG- and IFNGR1-Targeting Plasmids

The gRNA sequences targeting *IFNG* and *IFNGR1* were designed using the on-line CRISPR design tool, Cas-Designer (http://www.rgenome.net/cas-designer, accessed on 1 May 2020, version updated on December 2016, Bae Lab, Seoul National University College of Medicine, Seoul, Korea). Sequence details are provided in Supplementary Table S2. Original gRNA sequences were synthesized with slight modifications, including addition of guanine (G) at the first nucleotide position (to facilitate the activity of the U6 promoter [38]) and inclusion of restriction-enzyme site overhangs. Each gRNA cassette was inserted into the integrated CRISPR-Cas9 vector using restriction-enzyme sites.

### 4.3. Cell Culture and Transfection

The Vero cell line (African green monkey kidney epithelial cells) was kindly provided by Dr. Young Bong Kim (Konkuk University, Seoul, Korea). Cells were maintained in Dulbecco’s modified Eagle’s medium (DMEM) (Capricorn Scientific, Ebsdorfergrund, Germany) and supplemented with 10% heat-inactivated fetal bovine serum (FBS) and 1% penicillin–streptomycin at 37 °C in a humidified 5% CO_2_ incubator. For plasmid transfection, Vero cells were grown to ~90% confluence in a 12-well plate and then transfected with 1 μg of *IFNG*- or *IFNGR1*-targeting integrated CRISPR-Cas9 plasmid using Lipofectamine 3000 (Thermo Fisher Scientific, Inc., Waltham, MA, USA) at a ratio of 1:2.5 (plasmid:Lipofectamine 3000), in accordance with the instructions of the manufacturer. After 72 h, the transfection efficiency was analyzed by counting GFP-positive cells by fluorescence microscopy (Nikon Eclipse Ts2R, Nikon, Tokyo, Japan).

### 4.4. Single Cell Isolation

Seventy-two hours post-transfection, cells were harvested by trypsinization and diluted to a concentration of 100 cells per 100 μL media. Single-cell clones were isolated by seeding cells at a two-fold serial dilution in 96-well culture plates. Cells were grown at 37 °C in a humidified 5% CO_2_ incubator until they reached ~80% confluence. Each cell clone was further propagated in a 24-well plate for PCR-based genotyping.

### 4.5. PCR-Based Genotyping of Knockout Cell Lines

Single-cell clones were obtained from 24-well plates by trypsinization, and genomic DNA was extracted using a Wizard Genomic DNA Purification Kit (Promega, Madison, WS, USA), in accordance with the protocol of the manufacturer. Gene editing was examined by PCR-based genotyping using primers targeting *IFNG* or *IFNGR1* (Supplementary Table S3). PCR products were separated according to size using gel electrophoresis, and deleted sequences were investigated by Sanger sequencing (Supplementary Table S4). Detailed information on PCR primers and sequencing primers is provided in Supplementary Tables S3 and S4.

### 4.6. Quantitative Reverse Transcription-Polymerase Chain Reaction (RT-qPCR)

Total RNA was isolated from wild-type or knockout cell lines using a PureLink RNA Mini Kit (Invitrogen, Carlsbad, CA, USA), in accordance with the instructions of the manufacturer. The quantity and quality of isolated RNAs were analyzed by UV spectrophotometry (NanoPhotometer N60/N50; Implen, München, Germany). cDNA was synthesized from total RNA using SuperScript III first-strand synthesis supermix (Invitrogen, Carlsbad, CA, USA), and qPCR was performed on a LightCycler 96 system (Roche, Mannheim, Germany) using SsoAdvanced Universal SYBR Green Supermix (Bio-Rad, Hercules, CA, USA). Gene-specific primers used for qPCR are listed in Supplementary Table S5. Expression levels of genes were normalized to those of glyceraldehyde-3-phosphate dehydrogenase (*Gapdh*).

### 4.7. Viruses

HCoV-OC43 and HSV-1 were purchased from the Korea Bank for Pathogenic Viruses (KBPV; Seoul, Korea). HCoV-OC43 was propagated in Vero E6 cells in DMEM supplemented with 2% FBS and 1% penicillin–streptomycin at 37 °C in a humidified 5% CO_2_ environment. HSV-1 was amplified in Vero cells grown in DMEM supplemented with 5% FBS and 1% penicillin–streptomycin at 37 °C in a humidified 5% CO_2_ incubator. All experiments were performed in a Biosafety Level 2 facility.

### 4.8. Virus Infection and Monitoring of Cytopathic Effects

Wild-type and knockout cell lines were seeded into T25 flasks and grown until they reached ~80% confluence. The next day, the medium was removed, and cells were washed with serum-free media. Cells were then infected with 1 mL of HCoV-OC43 or HSV-1 (MOI = 0.1) and incubated for 1 h at 37 °C in a CO_2_ incubator. While incubating, flasks were gently rocking by hand at 15-min intervals. After 1 h, serum-containing medium was added to each flask to achieve a final FBS concentration of 2% for HCoV-OC43 and 5% FBS for HSV-1. Infected cells were further incubated for 3 days with HSV-1 and 4 days with HCoV-OC43. Cytopathic effects were monitored daily with a light microscope (Nikon Eclipse Ts2, Nikon, Tokyo, Japan) using a 40× objective lens; photographs of cell monolayers were captured every 24 h post infection.

### 4.9. Virus Quantification by Endpoint Dilution Assay

Every 12 h post infection, 100 µL of infected media was obtained and stored at −80 °C. The 50% tissue culture infectious dose (TCID_50_) for each sample was determined by seeding Vero cells in 96-well plate and incubating for 24 h. The next day, an HSV-1 or HCoV-OC43 virus sample was diluted 10-fold and prepared as 5-fold serial dilutions. Confluent cell monolayers were washed once with serum-free media, and 100 μL of diluted virus samples was applied to each well. Cells infected with HSV-1 were incubated for 4 days, and those with HCoV-OC43 were incubated for 5 days. Cytopathic effects were monitored using a light microscope. Culture plates were stained with crystal violet and virus titers were determined based on TCID_50_, according to the Reed and Muench endpoint method.

### 4.10. MTT Assay

Wild-type and knockout cells were seeded into 96-well plates and grown until reaching ~90% confluence. After removing the medium, cells were infected with HSV-1 or HCoV-OC43 (MOI = 0.1) and incubated for 1 to 3 days for HSV-1 and 1 to 4 days for HCoV-OC43. Each day, the medium was discarded and replaced with 100 µL of phenol red-free culture medium, after which 20 μL of 3-(4, 5-dimethylthiazol-2-yl) 2,5-diphenyl tetrazolium bromide salt solution (12 mM MTT stock solution) was added. After incubation at 37 °C for 4 h, 80 µL of medium was removed and 50 µL of DMSO was added to each well. After incubation at 37 °C for 10 min, absorbance was measured at 540 nm using a microplate reader (Epoch; BioTek, Winooski, VT, USA).

### 4.11. Statistical Analysis

Each graph is presented as means of independent replicates with standard deviation (SD). For statistical analysis, a two-tailed unpaired *t*-test was applied in case of a two-sample comparison by GraphPad Prism 9.4.0. (GraphPad Software, San Diego, CA, USA). For comparison of more samples, one-way non-parametric ANOVA (Kruskal Wallis test) was used to assess whether a difference is significant. The Shapiro–Wilk normality test was applied to verify the normal distribution of the data before statistical analysis.

## Data Availability

Any data or materials such as plasmid vectors or cell lines that support the findings of this study can be made available by the corresponding author upon request.

## Supplementary Materials

The supporting information can be downloaded at: https://www.mdpi.com/article/10.3390/ijms23158217/s1.
