# Supplementary material for: Integrated CRISPR-Cas9 System-Mediated Knockout of IFN-γ and IFN-γ Receptor 1 in the Vero Cell Line Promotes Viral Susceptibility
Source: Int J Mol Sci. 2022 Jul 26;23(15):8217. doi: 10.3390/ijms23158217 (PMC9368479; doi:10.3390/ijms23158217)
Supplement: Supplementary file 1 [file ijms-23-08217-s001.zip › ijms-1829530-supplementary.pdf]

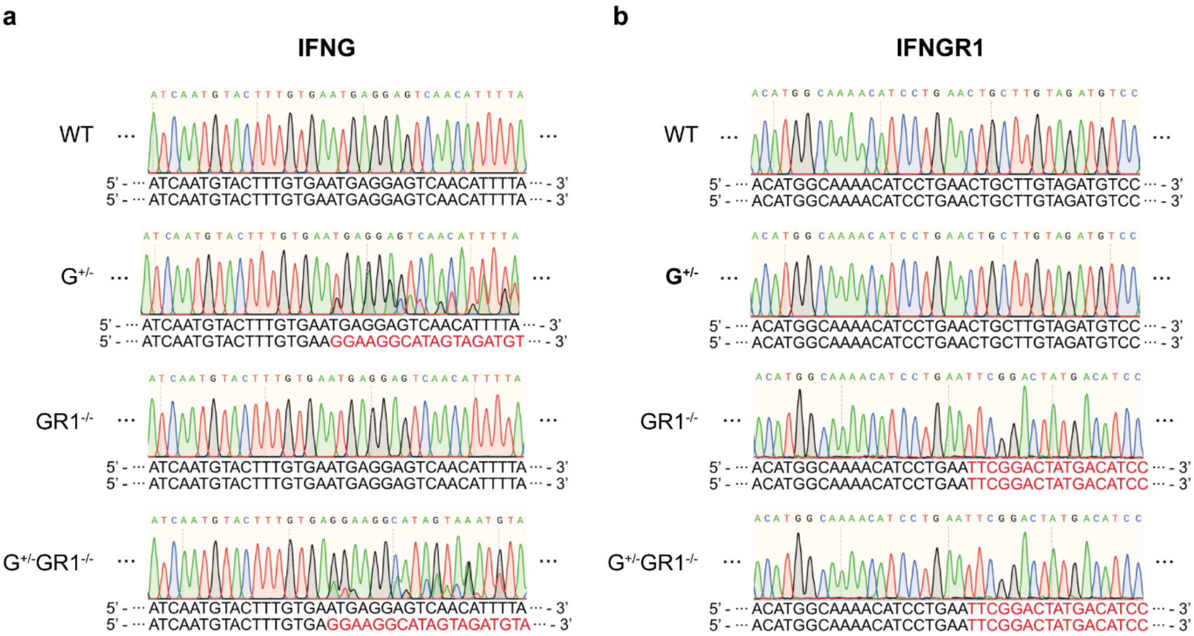

Supplementary Figure S1. Sanger sequencing analysis of *IFNG*- and *IFNGR1*-knockout Vero cell lines. (a) Sequencing results of the *IFNG* locus. WT, wild-type Vero cell line; G$^{+/-}$, heterozygous deletion of *IFNG* in the Vero cell line; GR1$^{-/-}$, homozygous deletion of *IFNGR1* in the Vero cell line; G$^{+/-}$GR$^{-/-}$, combined deletion of *IFNG* and *IFNGR1* in the Vero cell line. (b) Sequencing results of the *IFNGR1* locus in WT, G$^{+/-}$, GR$^{-/-}$, and G$^{+/-}$GR$^{-/-}$ cell lines.

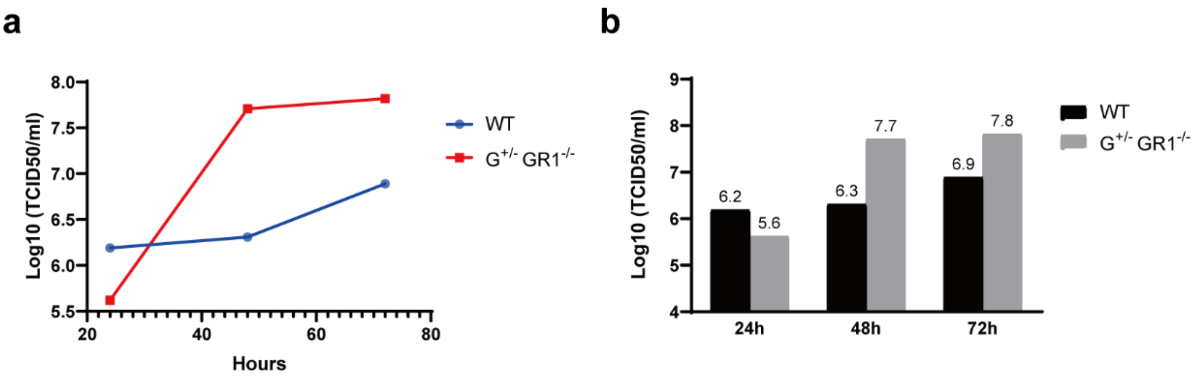

Supplementary Figure S2. Comparison of HSV-1 virus growth in the wild-type Vero cell line and *IFNG*/*IFNGR1*-knockout Vero cell line. (**a**) HSV-1 growth curves in the wild-type Vero cell line (WT) and *IFNG*/*IFNGR1*–double-knockout cell line ($G^{+/-}GR1^{-/-}$). Infectious titer was measured at the indicated time points in each cell line by endpoint dilution assay. (**b**) Bar graph showing $TCID_{50}$ values for HSV-1 measured at 24-hour intervals.

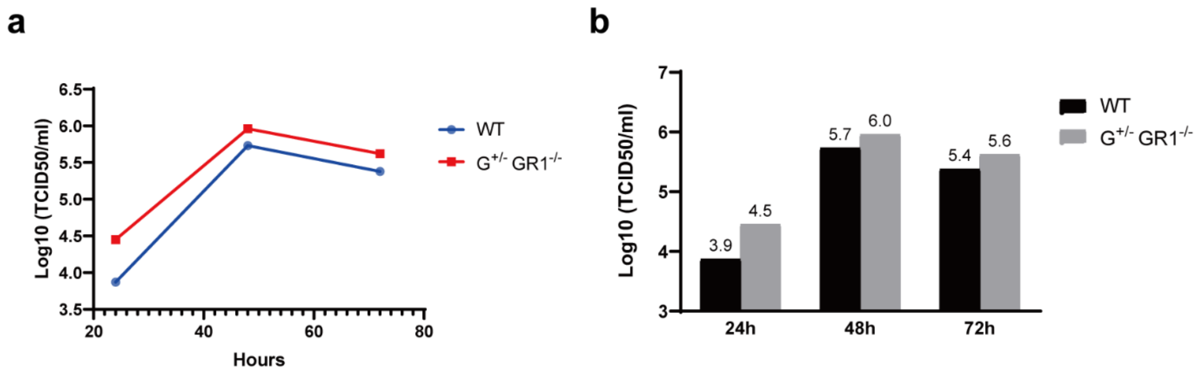

Supplementary Figure S3. Comparison of HCoV-OC43 virus growth in the wild-type Vero cell line and *IFNG*/*IFNGR1*-knockout Vero cell line. (**a**) HSV-1 growth curves in the wild-type Vero cell line (WT) and *IFNG*/*IFNGR1*–double-knockout cell line ($G^{+/-}GR1^{-/-}$). Infectious titer was measured at the indicated time points in each cell line by endpoint dilution assay. (**b**) Bar graph showing $TCID_{50}$ values for HCoV-OC43 measured at 24-hour intervals.

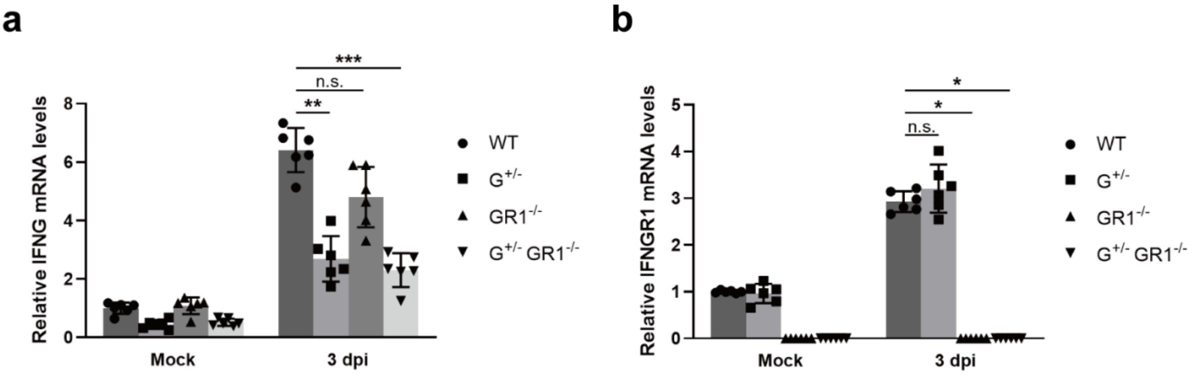

Supplementary Figure S4. Relative expression levels of *IFNG* and *IFNGR1* after HCoV-OC43 infection in *IFNG*- and *IFNGR1*-knockout cell lines. (**a**) Relative expression levels of *IFNG* in $G^{+/-}$, $GR1^{-/-}$, $G^{+/-}GR1^{-/-}$ cell lines. *IFNG* mRNA levels were normalized to those of wild-type cells. Results are shown as means ± SD of six independent replicates (*$p < 0.05$, **$p < 0.01$, ***$p < 0.001$, *n.s.*, not significant). (**b**) Relative expression levels of *IFNGR1* in $G^{+/-}$, $GR1^{-/-}$, and $G^{+/-}GR1^{-/-}$ cell lines. *IFNGR1* mRNA levels were normalized to those of wild-type cells.

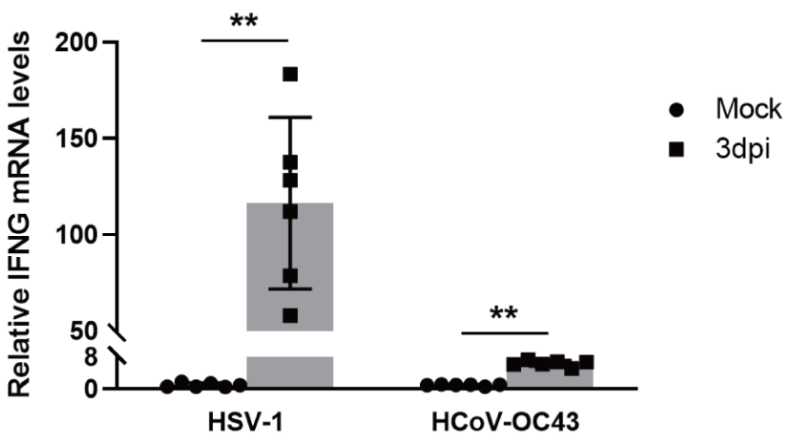

Supplementary Figure S5. Relative expression levels of *IFNG* after viral infection. *IFNG* mRNA levels of infected Vero cells were normalized to those of uninfected cells. *IFNG* mRNA levels were measured by RT-qPCR after 48 hours of HSV-1 infection, and 72 hours of HCoV-OC43, respectively. Results are shown as means ± SD of six independent replicates (**$p < 0.01$).

Supplementary Table S1. Deleted sequences in *IFNG*- and *IFNGR1*-knockout cell lines

**Genotype**	***IFNG* Sequences**	**Mutations**
Wild-type	5’ - ···CTTTGTGAATGAGGAGTC···TACACTACAGGAAGGCAT··· - 3’	
$G^{+/-}$	5’ - ···CTTTGTGAA−−−−−−−−−··−−−−−−−−−−GGAAGGCAT··· - 3’	1,405 bp deletion (*IFNG* Δexon1)
$GR1^{-/-}$	5’ - ···CTTTGTGAATGAGGAGTC···TACACTACAGGAAGGCAT··· - 3’	
$G^{+/-}GR1^{-/-}$	5’ - ···CTTTGTGA−−−−−−−−−−−··−−−−−−−−−−GGAAGGCAT··· - 3’	1,406 bp deletion (*IFNG* Δexon1)
**Genotype**	***IFNGR1* Sequences**	**Mutations**
Wild-type	5’ - ···CATCCTGAACTGCTTGTA···GATCCTGTTTTCGGACTAT··· - 3’	
$G^{+/-}$	5’ - ···CATCCTGAACTGCTTGTA···GATCCTGTTTTCGGACTAT··· - 3’	
$GR1^{-/-}$	5’ - ···CATCCTGAA−−−−−−−−−··−−−−−−−−−−TTCGGACTAT··· - 3’	1,651 bp deletion (*IFNGR1* Δexon3)
$G^{+/-}GR1^{-/-}$	5’ - ···CATCCTGAA−−−−−−−−−··−−−−−−−−−−TTCGGACTAT··· - 3’	1,651 bp deletion (*IFNGR1* Δexon3)

Supplementary Table S2. gRNA sequences for targeting *IFNG* and *IFNGR1*

Target gene	gRNA sequences with PAM	Target region
IFNG	5'-GATCAATGTACTTTGTGAATG AGG-3'	Exon 1
	5'-GTGACGTAATACACTACAGGA AGG-3'	Exon 1 ~ Exon 2
IFNGR1	5'-GTGGACATCTACAAGCAGTTC AGG-3'	Exon 2 ~ Exon 3
	5'-GGATGTCATAGTCCGAAAAC AGG-3'	Exon 3 ~ Exon 4

Supplementary Table S3. Target-specific primers for PCR-based genotyping

Target gene	Primer sequences		PCR product size
IFNG	Forward	5’-CCTGTGTGGCTTGTATTATATTTC-3’	1,831 bp
	Reverse	5’-GTTCAACAAAGCTGATGATACTCC-3’	
IFNGR1	Forward	5’-GTACCAGATCATGCCACAGG-3’	2,061 bp
	Reverse	5’-ATGCAGACGGTGTGGTAATC-3’	

Supplementary Table S4. Target-specific primers for Sanger sequencing

Target gene	Primer sequences	
IFNG	Forward-1	5'-CCTGTGTGGCTTGTATTATATTTC-3'
	Forward-2	5'-CAAATGCCACAAAACCTTAG-3'
	Forward-3	5'-GGTGAGAACTCATTCAGATG-3'
	Reverse 1	5'-GTTCAACAAAGCTGATGATACTCC-3'
IFNGR1	Forward-1	5'-GTACCAGATCATGCCACAGG-3'
	Forward-2	5'-AGGCTGAGGCAGGAGAATGG-3'
	Forward-3	5'-TCTGATCACGTTGGTGATCC-3'
	Reverse-1	5'-ATGCAGACGGTGTGGTAATC-3'

Supplementary Table S5. Gene-specific primers for RT-qPCR analyses

Target gene	Primer sequences	
IFNG	Forward	5'-TGGAAAGAGGAGAGTGACAG-3'
	Reverse	5'-TCAGCTTTTCGAAGTCATCC-3'
IFNGR1	Forward	5'-AACTATGGTGTTAAGAATGC-3'
	Reverse	5'-GGTCCAATTTTTCCATCTCG-3'
TNF-α	Forward	5'-GCTGCACTTTGGAGTGATCG-3'
	Reverse	5'-CCAGCTGGTTATCTGTCAGC-3'
IL-15	Forward	5'-CCCAGTTGCAAGGTAACAGC-3'
	Reverse	5'-TCCTCACATTCTTTGCATCC-3'
IRF1	Forward	5'-CCATTCACACAGGCCGATAC-3'
	Reverse	5'-GGAATCCCCACATGACTTCC-3'
IL-6	Forward	5'-AAGGAGACATGTAACAGGAG-3'
	Reverse	5'-CTAGGTATACCTCAAACTCC-3'
Nectin-1	Forward	5'-CTGCAGTATTAGTGTCCTCC-3'
	Reverse	5'-GTGCAGGATAAAGGAGATGC-3'
TLR2	Forward	5'-CGAATACACAGTGTAACAGG-3'
	Reverse	5'-GAAAAGAGTCAAGTTGCTCC-3'
TLR3	Forward	5'-GACAAACCTCACTATGCTCG-3'
	Reverse	5'-CCGTTTCAAATTCAGGTACC-3'
TLR7	Forward	5'-TGAGTCTCTTAGAACTCTGG-3'
	Reverse	5'-GGCATACCATCAAAAACTCC-3'
